# Antibodies towards TVLLPVIFF Amino Acid Sequence of TNF Receptor Induced by *Helicobacter pylori* in Patients with Coronary Heart Disease

**DOI:** 10.3390/jcm11092545

**Published:** 2022-05-01

**Authors:** Weronika Gonciarz, Agata Tomaszewska, Agnieszka Krupa, Tomasz Rechciński, Maciej Chałubiński, Marlena Broncel, Magdalena Chmiela

**Affiliations:** 1 Department of Immunology and Infectious Biology, Institute of Microbiology, Biotechnology and Immunology, Faculty of Biology and Environmental Protection, University of Łódź, Banacha 12-16, 90-237 Lodz, Poland; weronika.gonciarz@biol.uni.lodz.pl (W.G.); agata.tomaszewska@edu.uni.lodz.pl (A.T.); agnieszka.krupa@biol.uni.lodz.pl (A.K.); 2 Lodz Institutes of the Polish Academy of Sciences, The Bio-Med-Chem Doctoral School, University of Lodz, Banacha 12/16, 90-237 Lodz, Poland; 3 Clinic and Department of Cardiology, Medical University of Lodz, 92-213 Lodz, Poland; rechcinski@gmail.com; 4 Department of Immunology and Allergy, Medical University of Lodz, Pomorska 251, 92-213 Lodz, Poland; maciej.chalubinski@umed.lodz.pl; 5 Laboratory of Tissue Immunopharmacology, Department of Internal Diseases and Clinical Pharmacology, Medical University of Lodz, Kniaziewicza 1/5, 91-347 Lodz, Poland; marlena.broncel@umed.lodz.pl

**Keywords:** antigenic mimicry, *Helicobacter pylori*, human TNFR, atherogenesis

## Abstract

Background: Molecular mimicry between *Helicobacter pylori* (Hp) and the host components resulting in induction of cross-reacting antibodies has been suggested as accessory mechanism in the development of coronary heart disease (CHD). A potential target for antibodies induced during Hp infection by the components of these bacteria might be amino acid sequence TVLLPVIFF (P1) of tumor necrosis factor receptor (TNFR), which is exposed on vascular endothelium and immunocompetent cells, driving inflammation. Aim: To examine whether anti-P1 IgG are induced during Hp infection in CHD patients. Methods: Sera from CHD patients infected with Hp (54) vs. sera of uninfected healthy donors (22) were tested by the ELISA for anti-*H. pylori* antibodies, anti-P1 IgG, and for antibodies towards control sequence IAKEGFEKIS (P2). Sera of *Caviae porcellus* infected experimentally with Hp (30) or uninfected (10) were included into this study. The same serum samples, which were positive for anti-P1 IgG, were adsorbed with Hp and then subjected to the ELISA. The biological activity of anti-P1 IgG was assessed in complement (C1q) binding assay. Results: Sera of 43 CHD patients seropositive for anti-Hp IgG contained anti-P1 IgG binding C1q. Additionally, 10 serum samples of animals seropositive for anti-Hp IgG contained anti-P1 IgG. Anti-P1 IgG in tested sera were neutralized by their adsorption with Hp. Conclusion: In CHD patients infected with Hp, antibodies cross-reacting with TNFR common sequence are produced. Further studies are necessary to define immunogenic Hp determinants and to confirm possible cellular effects of cross-reacting antibodies.

## 1. Introduction

*Helicobacter pylori* are Gram-negative, microaerophilic rods that temporarily colonize the human oral cavity and then chronically colonize the human gastric mucosa (about 50% of the population); they can cause gastritis, duodenitis, ulcer disease, gastric cancer, and mucosa-associated lymphoid tissue lymphoma (MALT) [1,2]. Various virulence factors, including urease, numerous adhesins, vacuolating cytotoxin A (VacA), cytotoxin-associated gene A(CagA) protein, and other compounds, facilitate *H. pylori* colonization and survival in the stomach. They contribute to gastric tissue damage and development of local and systemic inflammation [3,4]. It has been suggested that *H. pylori* chronic infection may increase the risk of systemic disorders [5,6]. It might be due to induction by *H. pylori* that VacA, CagA, heat shock protein (Hsp), urease, or Lewis determinants of lipopolysaccharide (LPS) of antibodies potentially cross-react with the host components [7,8,9,10].

CHD is multifactorial vascular disease, which depends on individual predispositions and environmental factors: a particularly high-fat diet and possible microbial pathogens, which induce inflammation and immune effector mechanisms [11]. The role of the immune system is manifested by the presence of macrophages, T lymphocytes, and immunoglobulins within lesions [12]. However, up to now, it is impossible to identify a sub-group of CHD patients in whom infection modulates the atherogenesis.

Using an experimental model of *H. pylori* infection in *Caviae porecllus* fed with the high-fat diet, we showed that this infection is associated with induction of oxidative stress, local and systemic inflammation, in conjunction with an infiltration of vascular endothelium with inflammatory cells, diminished vascular elasticity, and development of proatherogenic environment synergistically with a high-fat diet [13]. The role of oxidized low-density lipoprotein (oxLDL) in atherogenesis has been reported [14,15,16,17,18]. Thus, oxidative stress elevated by *H. pylori* may drive oxidation of LDL. oxLDL is involved in vascular inflammation and penetration of monocytes due to upregulation of vascular cell adhesion molecule-1 (VCAM)-1, and intracellular adhesion molecule-1 (ICAM-1) deposition on endothelial cells [19].

Majority of CHD patients are exposed to *H. pylori* since they produce antibodies towards various *H. pylori* antigens [7,18,19,20]. However, data that do not confirm this correlation are also available [21,22]. Several studies have demonstrated the presence of *H. pylori* molecular material in arterial tissues in CHD patients [23,24]. The correlation between infection caused by *H. pylori* strains possessing a CagA pathogenicity island (Cag PAI), and elevated anti-CagA antibody production in patients with CHD has been reported [7,25,26,27,28]. Franceschi et al. showed by the Western blotting that anti-CagA antibodies recognized cytoplasmic and nuclear antigens in smooth muscle cells within atherosclerotic plaques in CHD patients, which suggests the molecular mimicry background [8].

According to theory of antigenic mimicry, only certain sequences of bacterial proteins are similar to host proteins. However, even small amino acid sequences can stimulate antibodies cross-reacting with the host targets and induce deleterious effects due to activation of complement, blocking cell receptors or modulating signaling pathways.

Recently, it has been suggested that TNFR is involved in the development of atherosclerosis due to promotion of inflammatory responses [29,30]. Antibodies towards TNFR potentially might affect these processes.

In this study, we asked whether *H. pylori* components may induce in infected CHD patients the antibodies cross-reacting with the epitopes of TVLLPLVIFF amino acid sequence in TNFR. We tested sera from CHD patients infected with *H. pylori* vs. control sera of uninfected healthy donors by the enzyme-linked immunosorbent assay (ELISA) using antigenic complex of surface *H. pylori* proteins called a glycine acid extract (GE) and synthetic peptides: P1 peptide consisting of TNFR amino acid sequence (TVLLPLVIFF) and control P2 peptide containing neutral sequence (IAKEGFEKIS). To show that serum antibodies cross-reacting with the sequence of TNFR are induced by *H. pylori* components, we also used a *Caviae porcellus* model experimentally infected with these bacteria. *Caviae porcellus* is sensitive to *H. pylori* infection and develops inflammatory and immune responses towards *H. pylori*, which might be evaluated on the basis of the selected histological and an immune markers as in humans [31,32].

In the current study, the same serum samples before and after adsorption with the reference *H. pylori* strain were examined for anti-P1 IgG in the ELISA. Biological activity of anti-P1 antibodies was assessed by the ability to bind C1q subunit of complement (ELISA).

## 2. Materials and Methods

### 2.1. Patients and Controls

The patients (54, both sexes, mean age 59.7 ± 7.4, males 65.3%) with CHD confirmed by standardized coronarography were from the 2nd Cardiology Clinic of Bieganski Regional Hospital (Medical University of Lodz, Lodz, Poland) and the Department of Internal Diseases and Clinical Pharmacology (Medical University of Lodz, Lodz, Poland). The inclusion criteria were the classic CHD risk factors: arterial hypertension (intake of antihypertensive drugs prior to enrolment, systolic blood pressure > 140, or diastolic blood pressure > 90 mm Hg), diabetes (glucose-reducing agents or insulin intake prior to enrolment), fasting glucose level (>126 mg/dL (7 mmol/L) double checked), glycosylated hemoglobin (Hb) level (>7% or positive oral glucose tolerance test with 75 g glucose), dyslipidemia (hypolipidemic agents intake prior to enrolment), hyperlipidemia: total cholesterol level (>190 mg/dL (5.0 mmol/L), LDL cholesterol level (>115 mg/dL (3.0 mmol/L)), high-density lipoprotein (HDL) cholesterol (< 40 mg/dL (1.0 mmol/dL)), or triglycerides (>150 mg/dL (1.2 mmol/L), obesity-body mass index (BMI) (>32), and nicotinism. Healthy adults with negative history of cardiovascular disease (48, both sexes, mean age 56.5 ± 5.2, males 63.6%) were from one of the basic health care units in Lodz. This group was selected based on the exclusion of classis risk factors for CHD (arterial hypertension, hyperlipidemia, diabetes, smoking, obesity). For experiments, 22 healthy donors truly negative for *H. pylori* were selected based on negative ^13^C urea breath testing and negative serum anti-*H. pylori* antibodies [33]. All participants signed the informed consent, with the privacy guarantee. The study was approved by the Local Ethics Committee. Blood samples were collected in a fasting state on admission before the medical and pharmacological intervention. The routine laboratory testing, as indicated in inclusion criteria, was performed using automatic equipment (Cobas 6000, Roche Diagnostic, G Serum) from the same blood sample was obtained within 1h by 30 min incubation at room temperature and 30 min incubation at 4 °C, followed by centrifugation (2000× *g*, 10 min, 4 °C). The sera were aliquoted and stored at −80 °C and thawed directly before experiments.

### 2.2. H. pylori Infection in Caviae porcellus

An in vivo model of *H. pylori* infection in *Caviae porcellus* was developed as previously described [32,34,35], according to the European Union (EU) directive (Directive 2010/63/EU) and the Council low (Dz.U. L 276 z 20.10.2010, s. 33–79) and approved by the Local Ethics Committee (LKE9), Medical University of Lodz, Poland, which was established by the Ministry of Science and Higher Education in Poland (Decision 58/ŁB45/2016). Three-month old, 400–600 g of weight male Himalayan *Caviae porcellus* (previously guinea pig) were bred in the Animal House (Faculty of Biology and Environmental Protection, University of Łódź, Lodz, Poland), kept in cages with free access to water, and fed with standard chow. The animals were inoculated *per os* three times (at two-day intervals) with 1 mL of Brucella broth (control) or 1 mL fresh suspension of *H. pylori* CagA+ strain CCUG 17874 (Culture Collection University of Gothenborg, Gothenburg, Sweden), 1 × 10^10^ colony forming units-CFU/mL. Before inoculation, the animals were given orally 1 mL of 0.2 M NaHCO_3_ to neutralize stomach pH. In total, 40 animals were used: 10 uninfected (control) and 30 with *H. pylori* infection lasting 7 days (10) or 28 days (20). Stool and blood samples as well as gastric tissue (after euthanasia) were collected in the above time points.

### 2.3. H. pylori Status

*H. pylori* status in patients and healthy donors was estimated by the ^13^C urea breath testing (^13^C UBT) [33] and the enzyme-linked immunosorbent assay (ELISA) for IgG towards *H. pylori* antigenic complex called a glycine acid extract (GE), as previously described [34]. *H. pylori* status and inflammatory response in animals were confirmed by microbiological (culture of bacteria), molecular (detection of the *ureC* and *cagA* by the polymerase chain reaction (PCR), histological (detection of *Helicobacter*-like organisms-HLO, infiltration of granulocytes and lymphocytes) examination, and by detection of *H. pylori* antigens in stool samples (Immundiagnostik AG, Benshei, Germany), as previously described [32,34]. Coincidence between diagnostic test was 98%.

### 2.4. Synthetic Peptides

The P1 peptide with the amino acid sequence (aa sequence: TVLLPLVIFF), identified in human or *Caviae porcellus* TNFR by the Basic Local Alignment Search Tool (BLAST), or P2 peptide without this sequence (aa sequence: IAKEGFEKIS) were synthesized by Lipopharm, Gdańsk, Poland.

### 2.5. Adsorption of Sera

Selected serum samples from humans or *Caviae porcellus* that were positive for anti-P1 IgG were absorbed twice with the heat-killed (1 h, 80 °C) *H. pylori* CCUG 17874 as previously described [36]. The same non-adsorbed or adsorbed serum samples were used for detection of anti-P1 IgG in the ELISA.

### 2.6. ELISA

The GE, the antigenic complex from the reference *H. pylori* CCUG 17874 (10 µg/mL), and P1 or P2 (100 ng/mL), all in carbonate buffer (pH 9.6), were applied to the wells of 96-well microtiter plate (100 μL/well) for 18 h, at 4 °C. The wells were emptied, washed 3× in PBS with 0.05% Tween 80 (PBS/Tween, 250 μL/well), blocked with 3% bovine serum albumin (BSA) in PBS/Tween (300 μL/well) for 2 h at room temperature, and washed 5 times. Human (1:25) or animal sera (1:50), diluted in 1% BSA/PBS/Tween, were added to the wells (100 μL/well). In some experiments, the same serum samples non-adsorbed or adsorbed with heat-killed *H. pylori* (80 °C, 1 h) were used. The plates were incubated at 37 °C, 1 h, and washed. The secondary antibodies towards human or guinea pig IgG, labeled with horseradish peroxidase (HRP) (Dako, Glostrup, Denmark), 1:6000 or 1:4000, respectively, were added to the wells (100 µL/well) for 1 h at 37 °C. After washing, a staining solution of 1 mg ortho-phenylenediamine (OPD)/ml phosphate-citrate buffer, pH 5.0, and containing 0.5 μL/ml 30% H_2_O_2_ was added (100 μL/well). The reaction was developed for 20 min in the dark and stopped with 0.5 M citric acid (50 μL/well). The absorbance was measured at 450 nm using the Victor 2 reader (Wallac, Turku, Finland). The cut-off optical density (OD) 450 was determined for human sera (0.22 for IgG anti-P1/P2; 0.21 for IgG anti-GE) and animal sera (0.23 for IgG anti-P1/P2; 0.23 for IgG anti-GE) vs. ODλ450 for control wells coated with the antigens and developed with HRP- antibodies (ODλ450 = 0.25).

### 2.7. C1q Binding with P1-Anti-P1 IgG Immune Complexes

The P1 (100 ng/mL) in carbonate buffer (pH 9.6) was applied to the wells of 96-well plates (100 μL/well) for 18 h, at 4 °C. After washing with PBS/Tween, the wells were blocked with 3%BSA/PBS/Tween for 2 h at room temperature, and washed 5 times. Selected guinea pig sera positive for anti-P1 IgG, diluted 1:50 in 1% BSA/PBS/Tween, were added to the wells (100 μL/well) for 1 h at 37 °C. After washing, 100 µL fresh mouse serum (complement) was added for 1 h at 37 °C. Emptied wells were supplemented with the HRP-secondary antibodies towards mouse C1q (Santa Cruse Santa Cruz Biotechnology, Dallas, TX, USA), 1:200 (100 µL/well). After 1 h at 37 °C, the plates were washed and treated with the staining solution as previously. The color reaction was developed, and when stopped, the absorbance was measured at 450 nm using the Victor 2 reader.

### 2.8. Statistical Analysis

Differences between the two groups were assessed by the U Mann Whitney test and for more than two groups by the Kruskal Wallis ANOVA. *p*-value < 0.05 was significant. Data are presented as mean ± standard deviation (SD) or median ± range. For statistical analysis the STATISTICA 13 PL software was used (Stat Soft, Kraków, Poland).

## 3. Results

### 3.1. Anti-H. pylori GE IgG as Well as Anti-P1 or Anti-P2 IgG in Human Sera

**Figure 1:**
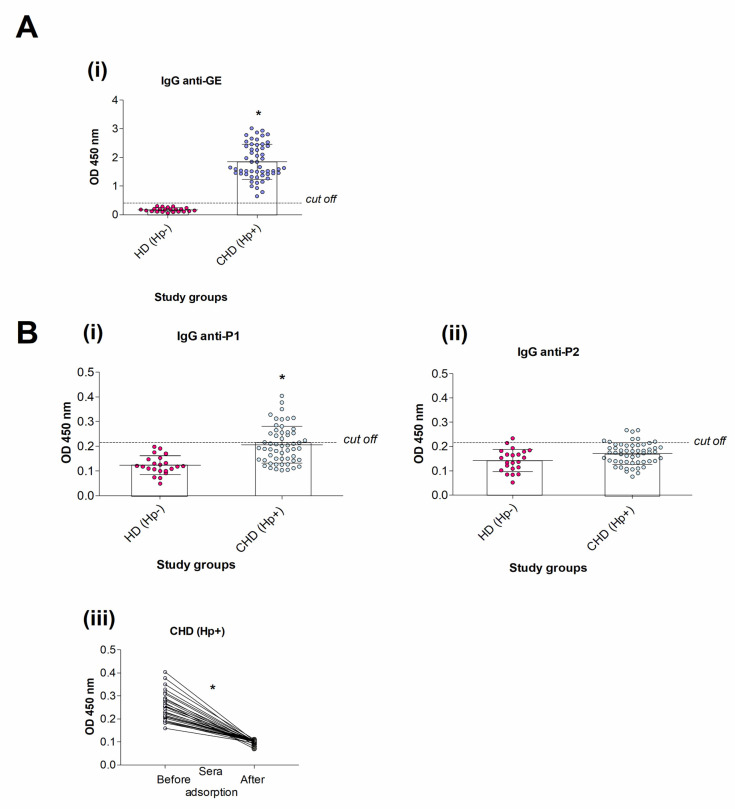
The prevalence and the level of antibodies in human sera. (**A**) IgG antibodies towards *H. pylori* glycine extract (GE) (**i**) Healthy donors (HD) seronegative for anti-GE IgG–HD Hp- (n = 22), patients with coronary heart disease (CHD) seropositive for anti-GE IgG–CHD Hp+ (n = 54); (**B**) anti-P1 IgG (**i**) and anti-P2 IgG (**ii**); the level of anti-P1 IgG before and after the adsorption of sera from CHD Hp+ patients with heat-inactivated *H. pylori* (**iii**). Shown are mean values ± standard deviation (SD). P1, synthetic peptide with the amino acid sequence (TVLLPLVIFF) present in human tumor necrosis factor receptor (TNFR); P2, control peptide (IAKEGFEKIS). The dot in the figure represents an individual data. Statistical significance: CHD(Hp+) vs. HD (Hp-) (Ai,Bi), CHD(Hp+) sera before vs. sera after adsorption with *H. pylori*, * *p* < 0.05.

The prevalence and the levels of anti-GE IgG and anti-P1/P2 IgG in tested human sera are shown in Figure 1. Sera (22) from adult healthy donors (HD), which did not contain anti-GE (OD = 0.189 ± 0.092), were defined as *H. pylori*-negative and were used as negative control vs. sera from CHD patients. All CHD patients (54) produced anti-GE IgG (Figure 1A(i)). Anti-P1 were not found in the sera of healthy donors (Figure 1B(i)) but were detected in 43/54 sera of CHD patients (Figure 1B(i)). Very low anti-P2 IgG level was detected in 1 serum sample of HD group and eight serum samples of CHD group (Figure 1B(ii)).

In order to link anti-P1 IgG production with *H. pylori* infection, we examined the presence of anti-P1 IgG in selected serum samples from CHD before and after the adsorption of serum samples with heat-killed *H. pylori.* The level of anti-P1 IgG was significantly diminished after the adsorption of sera with *H. pylori* (Figure 1B(iii)).

### 3.2. Specificity of Antibodies Raised in Response to Experimental H. pylori Infection in Caviae porcellus

**Figure 2:**
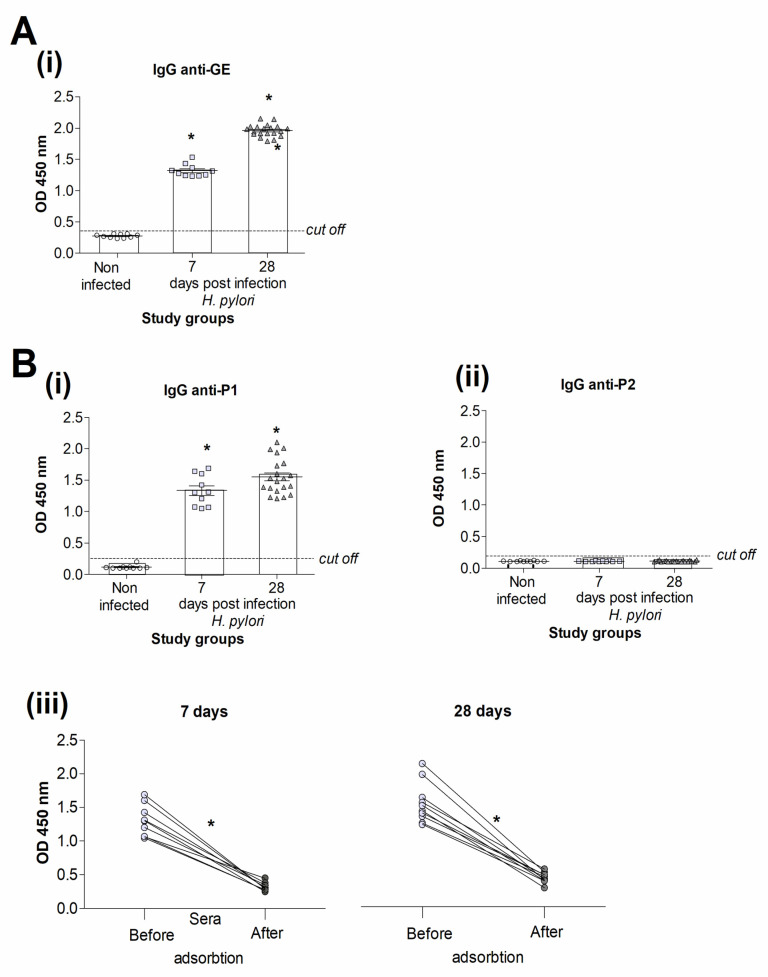
The prevalence and the level of antibody production in *Caviae porcellus* model. In *Caviae porcellus* infected experimentally with *H. pylori,* the enzyme-linked immunosorbent assay (ELISA) was used to examine serum samples for anti-GE IgG (**Ai**) and anti-P1/P2 IgG (**Bi**,**Bii**). Anti-P1 antibodies in serum samples of *H. pylori* infected animals, 7 and 28 days from inoculation, non-adsorbed or adsorbed with these bacteria (**Biii**). Sera were collected from control animals noninfected with *H. pylori*, n = 10, and from animals infected with *H. pylori*, 7 days, n = 10 or 28 days, n = 20, after the last inoculation with *H. pylori*. Shown are mean values ± standard deviation (SD). P1, synthetic peptide with the amino acid sequence (TVLLPLVIFF) of *Caviae porcellus* tumor necrosis factor receptor (TNFR); P2, control synthetic peptide (IAKEGFEKIS). GE, glycine acid extract from the reference *H. pylori* strain. The dot, square or triangle in the figure represent an individual data. Statistical significance: sera from *Caviae porcellus* infected with *H. pylori* vs. sera from non-infected animals (**Ai**,**Bi**,**Biii**) sera before and after adsorption * *p* < 0.05.

*Caviae porcellus* infected with *H. pylori* developed chronic gastritis related to infiltration of gastric mucosa with granulocytes, monocytes, and lymphocytes, which was confirmed by histological examination of gastric tissue specimens as shown previously [32,34]. In all animals infected with *H. pylori,* anti-GE IgG were produced after 7 and 28 days from inoculation. The level of such antibodies was higher after 28 than 7 days of infection (Figure 2A(i)). There was no anti-GE IgG in the sera of uninfected animals (Figure 2A(i)).

Anti-P1 IgG were detected by the ELISA (using P1) in the sera of all *H. pylori*-infected animals 7 and 28 days from the last inoculation but not in control animals (Figure 2B(i)). Animals noninfected with *H. pylori* or animals infected with these bacteria were free of anti-P2 antibodies to control peptide (Figure 2B(ii)). We confirmed the role of *H. pylori* in driving anti-P1 antibodies by development of the ELISA with P1 after the adsorption of serum samples with heat-killed *H. pylori.* The level of anti-P1 IgG was significantly diminished after the adsorption of the sera with *H. pylori* (Figure 2B(iii)).

### 3.3. Biological Activity of Anti-P1 Antibodies in Caviae porcellus Sera Assessed by C1q Binding Assay

**Figure 3:**
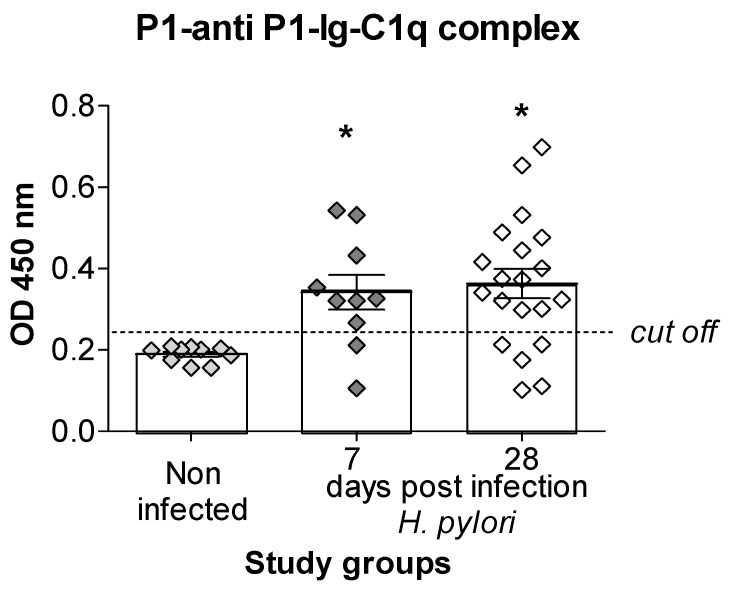
Detection of anti-P1-antibodies, which bind C1q subunit of complement, by the ELISA in serum samples of *Caviae porcellus* infected with *H. pylori.* Serum samples were added to microtiter plates coated with P1 peptide, and after binding with anti-P1 antibodies, the plates were incubated with C1q. The P1-anti-P1 IgG-C1q complexes were detected using anti-C1q antibodies labeled with horseradish peroxidase (HRP). The sera were collected from control animals noninfected with *H. pylori*, n = 10, or infected with these bacteria, 7 days, n = 10 or 28 days, n = 20, after the last inoculation with *H. pylori*. Shown are mean values ± standard deviation (SD). P1, synthetic peptide containing the amino acid sequence (TVLLPLVIFF) of *Caviae porcellus* tumor necrosis factor receptor (TNFR). The square in the figure represents an individual data. Statistical significance: * *p* < 0.05.

Biological activity of anti-P1 IgG present in *Caviae porcellus* sera was estimated by the ability of P1-anti-P1 IgG immune complexes to bind C1q subunit of complement in the ELISA with ani-C1q antibodies labeled with horseradish peroxidase (HRP). Anti-P1 antibodies, which were present in 8 out of 10 sera from animals at 7 days after inoculation with *H. pylori* and in 15 out of 20 sera from animals at 28 days post infection, were able to bind C1q. Such antibodies were not detected in the sera of animals uninfected with *H. pylori* (Figure 3).

## 4. Discussion

The development of CHD depends on nonmodifiable factors (e.g., age, gender, genetic predisposition) and modifiable factors (e.g., increased blood pressure, hyperlipidemia, obesity) [37]. Numerous studies have linked atherosclerosis with bacterial pathogens, such as *Chlamydophila pneumoniae, Mycoplasma pneumoniae,* a d *H. pylori*, and viruses, such as Cytomegalovirus and herpes simplex [38,39,40]. Pathological processes in CHD are driven due to chronic inflammation in conjunction with the deposition of oxygenated cholesterol in the vascular endothelium [15,16,37]. The role of *H. pylori* in CHD has been considered in relation to systemic inflammation; increased level of LDL [14], lipopolysaccharide binding protein (LBP), and homocysteine [24,40]; and intima thickness [41] as well as induction of antibodies cross-reacting with the host components [7]. The inflammation processes associated with CHD or infection are both related to increased levels of acute phase proteins, enhanced deposition of vascular integrins, and elevated levels of proinflammatory cytokines, including TNF-α [42,43]

Recently atherosclerosis has been defined as chronic inflammatory disease with autoimmune components, such as autoantibodies and immune T lymphocytes, which exacerebrate the disease [17,44,45]. Kimura et al. reported that in CHD patients, there are increased levels of anti-oxLDL antibodies and memory T lymphocytes (CD45RO+), which respond to oxLDL by the production of inflammatory cytokines [45].

It has been shown that *H. pylori* components, including HspB, LPS, urease, or CagA, induce antibodies cross-reactive with human Hsp60 [7,10], Lewis X/Y determinants [46], type 1 chemokine receptor (CCRL1) [9], and cytoplasmic or nuclear antigens in smooth muscle cells within atherosclerotic plaques [8]. Recently, the study by Ninomiya et al. revealed that CagA may participate in development of atherosclerosis by inhibition of LDL uptake into cells by binding to LDL receptor, which may cause acceleration of LDL in plasma and thus hypercholesterolaemia [47].

Preliminary searching of databases indicated that *H. pylori* possess amino acid sequences similar to the sequence (TVLLPLVIFF) of human or guinea pig TNFR, a key signaling receptor on innate immune cells and vascular endothelial cells. Accumulating data suggest that TNF plays a pivotal role in vascular dysfunction due to driving the increased production of reactive oxygen species (ROS) [29].

Anti-TNFR antibodies may potentially drive pathological processes in vivo due to complement-dependent cytotoxicity or influence a TNFR signaling. However, it is not known if antibodies towards TNFR could be induced in the organism during infection with *H. pylori*.

We found that CHD patients in this study were all exposed to *H. pylori.* It has been suggested that infections with *H. pylori*, particularly those strains that possess a Cag PAI, corelated with the development of systemic inflammation and increased risk of atherosclerosis and CHD [48,49].

We examined serum samples of CHD patients exposed to *H. pylori* for the presence of antibodies towards TVLLPLVIFF sequence, which was synthesized and used in the ELISA as P1 peptide. The presence of anti-P1 cross-reacting antibodies was detected in 47% of CHD patients who were infected with *H. pylori*. This might be due to different antigenic composition of *H. pylori* strains causing infection. It is also possible that not all CHD patients infected with *H. pylori* are predisposed to respond by anti-P1 antibody production, which potentially might be due to differences in local and/or systemic inflammatory environment of these patients.

The anti-P1 IgG level in the sera of CHD patients infected with *H. pylori* was significantly diminished after adsorption of the same serum samples with heat-inactivated *H. pylori*, confirming that these antibodies were generated in response to *H. pylori.* Interestingly, antibodies reacting with P2 peptide were detected in a single serum sample from a healthy donor, which was negative for anti-*H. pylori* IgG, and in eight serum samples from CHD patients infected with *H. pylori*, suggesting that such antibodies might be induced by infectious agents different than *H. pylori.*

To confirm the correlation between *H. pylori* infection and the production of anti-P1 antibodies, we used the model of experimental *H. pylori* infection in *Caviae porcellus* previously characterized by us in terms of immune response [32].

We showed that all *Caviae porcellus* infected with *H. pylori* but not control animals responded by the production of anti-GE IgG to the higher level at 28 rather than 7 days from the last inoculation, which indicated that duration of infection played the important role in the development of specific antibody response. In all animals, we showed anti-P1 IgG. All tested sera did not react with control P2 peptide. Adsorption of sera from *H. pylori*-infected animals (7 or 28 days after inoculation) with heat-inactivated *H. pylori* significantly neutralized anti-P1 antibodies, confirming that such antibodies were induced in response to *H. pylori* infection.

In this study, anti-P1 IgG in 2 out of 10 *Caviae porcellus*, 7 days after inoculation with *H. pylori*, were seropositive for anti-P1 IgG and were able to bind C1q. However, in 5 out of 20 serum samples seropositive for anti-P1 IgG at 28 days from inoculation, results were negative. Our observation could be explained by the differences in the formation and the availability of anti-P1 IgG in the immune complexes for C1q binding. We used for the study the heat-inactivated sera, so blocking C1q binding in the ELISA due to autologous C1q was excluded.

The limitation of this study is that we do not know the effects of anti-TVLLPLVIFF antibodies in vivo. However, C1q binding with anti-P1 antibodies bound with P1 in the immune complexes indicated the ability of complement activation by such antibodies and thus lysis of cells bearing TNFR leading to upregulation of inflammatory response, imbalance between cell proliferation and apoptosis, and potentially intima thickness [50,51]. Anti-P1 antibodies, soluble or bound in immune complexes, could potentially modulate the TNFR driven signaling of inflammatory response or apoptosis and thus provide pro-atherogenic environment.

## 5. Conclusions

In CHD patients, during infection with *H. pylori*, components of these bacteria may induce the production of IgG antibodies cross-reacting with the TVLLPLVIFF sequence of host TNFR. Binding of C1q subunit of complement by such antibodies suggests that they might have biological activity in vivo. Further studies are necessary to show whether antibodies towards TVLLPLVIFF sequence of human TNFR induced in response to *H. pylori* components possess blocking or activating properties and whether they can target vascular TNFR.

## Data Availability

The data are not publicly available due to privacy restrictions.
